# Burden of Podoconiosis in Poor Rural Communities in Gulliso *woreda*, West Ethiopia

**DOI:** 10.1371/journal.pntd.0001184

**Published:** 2011-06-07

**Authors:** Getahun Alemu, Fasil Tekola Ayele, Takele Daniel, Christel Ahrens, Gail Davey

**Affiliations:** 1 SOS Children's Villages International, Addis Ababa, Ethiopia; 2 Center for Research on Genomics and Global Health, National Human Genome Research Institute, National Institutes of Health, Bethesda, Maryland, United States of America; 3 Addis Ababa Health Bureau, Addis Ababa, Ethiopia; 4 International Institute of Child Health, London, United Kingdom; 5 Brighton and Sussex Medical School, Brighton, United Kingdom; Ghana Health Service, Ghana

## Abstract

**Background:**

Podoconiosis is an environmental lymphoedema affecting people living and working barefoot on irritant red clay soil. Podoconiosis is relatively well described in southern Ethiopia, but remains neglected in other parts of the Ethiopian highlands. This study aimed to assess the burden of podoconiosis in rural communities in western Ethiopia.

**Methodology/Principal Findings:**

A cross-sectional study was conducted in Gulliso *woreda* (district), west Ethiopia. A household survey in the 26 rural *kebeles* (villages) of this district was conducted to identify podoconiosis patients and to measure disease prevalence. A more detailed study was done in six randomly selected *kebeles* to describe clinical features of the disease, patients' experiences of foot hygiene, and shoe wearing practice. 1,935 cases of podoconiosis were registered, giving a prevalence of 2.8%. The prevalence was higher in those aged 15–64 years (5.2%) and in females than males (prevalence ratio 2.6∶1). 90.3% of patients were in the 15–64 year age group. In the detailed study, 335 cases were interviewed and their feet assessed. The majority of patients were farmers, uneducated, and poor. Two-third of patients developed the disease before the age of thirty. Almost all patients (97.0%) had experienced adenolymphangitis (ALA - red, hot legs, swollen and painful groin) at least once during the previous year. Patients experienced an average of 5.5 ALA episodes annually, each of average 4.4 days, thus 24 working days were lost annually. The incidence of ALA in podoconiosis patients was higher than that reported for filariasis in other countries. Shoe wearing was limited mainly due to financial problems.

**Conclusions:**

We have documented high podoconiosis prevalence, frequent adenolymphangitis and high disease-related morbidity in west Ethiopia. Interventions must be developed to prevent, treat and control podoconiosis, one of the core neglected tropical diseases in Ethiopia.

## Author Summary

Podoconiosis is a chronic non-infectious disease resulting in below-knee swelling of the legs in bare-footed people living in red clay soil areas. It is an important and yet neglected problem in tropical Africa, central and south America, and north India. Podoconiosis can be prevented by consistently wearing shoes and washing feet. We aimed to assess the burden of the disease, to characterize features of the disease, and to describe foot hygiene and shoe wearing practice of patients in west Ethiopia. First, we did a survey of the 26 rural villages. We identified 1,935 podoconiosis patients, giving a prevalence of 2.8%. Podoconiosis was twice as prevalent in females as males. Second, we did a more detailed study among 335 patients in six randomly selected villages. We found that the majority of patients were farmers, uneducated, and poor. The disease developed before the fourth decade of life and the majority of patients became bed-ridden because of frequent attacks of red, hot legs and swollen and painful groin. Shoe wearing was limited mainly due to lack of money. We conclude that podoconiosis imposes a huge burden in west Ethiopia, and recommend that interventions be developed to prevent, treat and control the disease.

## Introduction

Podoconiosis (endemic non-filarial elephantiasis) is a non-infectious geochemical disease caused by exposure of bare feet to red clay soil derived from volcanic rock. It results in progressive bilateral swelling of the lower legs. Podoconiosis is mostly a disease of agrarian people who work barefoot, particularly on red clay soils of volcanic areas [1], [2], [3], [4]. Mineral particles, absorbed through the skin of the foot, are taken up into macrophages in the lower limb lymphatics and are thought to induce an inflammatory response in the lymphatic vessels, leading to fibrosis and obstruction of the vessel lumen [5].

Podoconiosis is widespread in highland areas of tropical Africa, Central America and northern India. It is considered to be a considerable public health problem in more than ten African countries including Uganda [6], Tanzania [7], Kenya [8], Rwanda, Burundi, Sudan, Ethiopia [9], Cameroon [1], [10], and Equatorial Guinea [11].

It is estimated that the total number of cases per country is highest in Ethiopia [12], [13], [14], [15]. In Ethiopia the basalt area covers more than 200,000 km^2^ which is approximately one-fifth of the land surface, and the fertility of the soil in such areas attracts an agricultural population of 20.5 million people [5]. Eleven million Ethiopians (18% of the population) are at risk through exposure to the irritant soil, and estimate based on prevalence data from an endemic area in southern Ethiopia suggests that between 500,000 and 1 million people are affected.

In Ethiopia most studies on podoconiosis have been conducted in Wolaita zone, southern Ethiopia. In Wolaita zone the prevalence of podoconiosis is over 5% [4], [16], and people with podoconiosis are half as productive as controls, costing the zone more than US$16million annually [17]. Furthermore, podoconiosis is one of the most stigmatizing health problems in the zone [18], [19], [20]. An epidemiological study in resettlement schemes of west Ethiopia showed that the prevalence of podoconiosis is higher in indigenous (9%) people than in settlers (5%). People from non-endemic areas develop disease on average 9 years after being moved to an endemic area [21]. Apart from this, there are no recent studies on podoconiosis in west Ethiopia in general and in West Wollega zone in particular where podoconiosis is common. The present study aims to determine the magnitude of podoconiosis in Gulliso *woreda* of West Wollega zone in terms of prevalence, socio-economic impact, incidence/prevalence of associated morbidities, and to assess the experience and perspectives of patients regarding prevention and treatment.

## Methods

### Ethics statement

Ethical approval for the study was granted by the Ethical Review Committee of the Oromia Regional Health Bureau. Informed verbal consent was obtained from the study participants before conducting the study. When children aged under 18 years (the legal age for giving consent for research in Ethiopia) were encountered, consent was obtained from their parents or guardians, and assent was obtained from children aged 12 or above. The use of verbal consent was approved by the ethical review commitee because the majority of the study participants cannot read and write. During and at the end of the survey, enumerators and health workers provided health education about foot hygiene and foot wear to prevent podoconiosis and treat early stages of the disease. Cases with advanced form of the disease were advised to visit the nearest health facility (Ayira Hospital) for advanced care and treatment.

### Study area

The study took place in Gulliso *woreda* (a government administrative unit, equivalent to a district), West Wollega zone, Oromia region of Ethiopia. The *Woreda* is located 500 km west of Addis Ababa, the capital city of Ethiopia, and has an altitude of 1,500–1,800 m above sea level. The population of the *woreda* is 69,856, of which 88.7% live in 26 rural *kebeles* and are subsistence farmers producing coffee as a cash crop.

For many decades the study population has had access to well functioning health services though hospital and clinics run by non-governmental organizations. The population has also had above-average primary and secondary education resulting in a relatively higher literacy rate than the national average for rural areas in Ethiopia.

### Study design and sampling

The present study is a cross sectional quantitative study conducted in two phases. The first part of the study involved a survey of all households in the 26 rural *kebeles* (the smallest administrative unit/village in Ethiopia) of Gulliso *woreda* to identify podoconiosis patients. The head of the household was asked whether any member of the household had podoconiosis. When a case was reported in the household, his/her sex, age, and marital status were recorded, and both feet were clinically examined. Following this, the second part of the study was conducted in 6 *kebeles* selected using the simple random sampling method. *Kebeles* were allocated random numbers that were written in pieces of papers. All papers were rolled and shuffled in a hat, and six were drawn. The selected *kebeles* were Eka, Hawate Sutchi, Jarso Lalo, Sage Guji, Saka Jirbi, and Moga Kobera. All 335 patients identified in these *kebeles* were included in the study population. A structured questionnaire was administered to the identified patients to explore demographics of patients, features of disease, and previous experiences and future perspectives of patients on prevention and treatment methods of podoconiosis.

### Data collection

The first part of the study was conducted by 26 health extension workers in government service in Gulliso *woreda*. The researchers trained the health extension workers on the nature, aetiology, treatment and prevention of podoconiosis. Practical training was also provided on clinical diagnosis and disease staging using a recently developed podoconiosis staging system [22]. A pre-test done in Gulliso town to test the skills of the health extension workers ensured reliability of their diagnostic skills. Following this, the health extension workers registered people with podoconiosis in their respective *kebeles* over a period of two weeks. In the second part of the study seven nurses led by a member of the research team administered a structured questionnaire to podoconiosis patients in the 6 selected *kebeles*.

Clinical characteristics included (i) disease onset; (ii) adenolymphangitis (ALA) defined as painful inflammation of the foot and leg with swollen lymph nodes and fever [14], and its consequence on patients' health seeking behaviour and morbidity; and (iii) clinical assessment of legs and feet of patients with regard to presence of mossy lesions (fluid filled or papylomatous horny lesions giving the skin a rough appearance [22]) and wounds. The largest circumference of the leg was measured using a tape to a precision level of the nearest centimeter between the level of the ankle and knee.

### Data analysis

Data were entered and analysed using the Statistical Package for Social Sciences (SPSS) software v.10.0. Descriptive statistics was done using summary statistics such as frequency, mean/median, and summary figures and tables. The overall prevalence of podoconiosis was calculated as the ratio of the number of patients with podoconiosis and the total population in the surveyed *kebeles*. Statistical significance was tested using the Chi square test. The level of significance was set at α of 0.05.

## Results

### Study population and disease related characteristics

Table 1 illustrates characteristics of podoconiosis affected individuals identified in the survey. Of 1,935 patients, 659 (34.1%) were male, and the male to female prevalence ratio was 1∶2.6, showing podoconiosis to be more than twice as prevalent among females as males. The age distribution of cases shows that 90.3% of the cases belonged to the economically productive age group (χ^2^ = 31.7, p<0.001).

**Table 1:** Characteristics of the surveyed population of podoconiois patients (n = 1,935).

Characteristic	Category	Male (n = 659)	Female (n = 1,276)	Overall (n = 1,935)
Average age in years	Mean (s.d.)	43.4 (15.9)	39.3 (15.3)	40.7 (15.6)
	Range	14–90	6–95	6–95
Age distribution, n (%)	<15	2 (0.3)	10 (0.8)	12 (0.6)
	15–64‡	563 (85.6)	1182 (92.7)	1745 (90.3)
	>64	93 (14.1)	83 (6.5)	176 (9.1)
Marital status for those aged 18 years or above, n (%)	Never married	88 (13.7)	234 (19.2)	322 (17.3)
	Others	555 (86.3)	984 (80.8)	1539 (82.7)
Prevalence by age group,% (95% CI)	<15	0.02(0–0.04)	0.08(0.03–0.13)	0.05(0.02–0.08)
	15–64	3.5 (2.1–4.9)	6.8(5.4–8.2)	5.2(4.2–6.2)
	>64	2.3(1.5–3.1)	3.2(2.1–4.3)	2.7(2.0–3.4)
Prevalence by type of *kebele*, % (95% CI)	Settler#	1.5(0.6–2.4)	1.3(0.7–1.9)	1.4(0.9–1.9)
	Non-settler	1.5(0.5–2.5)	3.6(2.5–4.5)	2.8(2.1–3.6)
	Overall§	1.4(0.5–2.3)	3.6(2.6–4.6)	2.8(2.1–3.5)

**‡:** The economically active age group is defined as the population in the age group 15–64 years that is potentially economically active [23], [24].

# Settler *kebeles* are Saka Jirbi and G/Ganka.

**§:** Prevalence of podoconiosis was calculated as the ratio of number of patients with podoconiosis and the total population in the surveyed 26 *kebeles* of Gulliso *Woreda*.

The overall prevalence of podoconiosis was 2.8% (1,935/69,465) (95% CI = 2.1% to 3.5%). The prevalence was even higher among the economically active age group of 15–64 years (5.2%). The prevalence was also higher in kebeles with non-settler population than those with recent settler population (2.8% vs. 1.4%, χ^2^ = 14.79, p<0.001). The mean age of patients at the time of survey was 40.7 years. Of patients aged 18 years and above, 17.3% were never married.

Of 1,935 patients identified in the survey, 335 (17.3%) were approached for the detailed study in six randomly selected *kebeles*. Characteristics of the respondents are presented in Table 2. The detailed study subjects consisted of 243 (72.5%) females and 92 males (27.5%), giving a male to female ratio of 1∶2.6. About 85% of the patients belonged to the economically active age group of 15–64 years. More than half of the patients had no formal education. The proportion of uneducated females was higher than that of males, and the difference was statistically significant (χ^2^ = 9.08, p = 0.003). The majority (80.6%) of the study participants were farmers.

**Table 2:** Characteristics of the study population that were interviewed, n = 335.

Characteristic	Category	Male (n = 92)	Female (n = 243)	Overall (n = 335)
Average age in years	Mean (±s.d.)	49.6 (17.7)	40.5 (19.9)	43.0 (18.3)
	Median(range)	48 (15–98)	38 (8–89)	40 (8–98)
Age distribution in years, n (%)	<15	0(0.0)	3 (1.2)	3 (0.9)
	15–64	71 (77.2)	212 (87.2)	283 (84.5)
	>64	21 (22.8)	28 (11.5)	49 (14.6)
Read and write	Yes	54 (58.7)	98 (40.3)	152 (45.4)
	No	38 (41.3)	145 (59.7)	183 (54.6)
Level of education completed (for those that can read and write)	4th grade or below	33 (61.1)	57 (58.2)	90 (59.2)
	Grade 4–8	19 (35.2)	36 (36.7)	55 (36.2)
	Grade 9–10	1 (1.9)	4 (4.1)	5 (3.3)
	College and above	1 (1.9)	1 (1.0)	2 (1.3)
Occupation	Farmer	59 (83.1)	169 (79.7)	228 (80.6)
	Daily laborer	7 (9.9)	19 (9.0)	26(9.2)
	Others§	5 (7.0)	24 (11.3)	29 (10.2)

**§:** Others include- retired (2.4%); student (2.4%); collecting fire wood (2.1%); begging (1.5%).

### Features of disease

The average age of onset of podoconiosis was 26 years, and the average duration of illness between time of onset and time of interview was 17 years. Forty percent said the disease started before age 20 and two-thirds said it started before age 30. There was no statistical difference between the sexes with regard to onset of podoconiosis.

The majority of patients (325, 97%) had experienced adenolymphangitis at least once during the one year period preceding the date of interview. Patients reported an average of 5.5 such episodes/year and 96% said they had to stay in bed (mean 4.4 days/episode); meaning that on average each patient lost 24 days of activity per year (range, 0–192 days per year). During episodes of adenolymphangitis, the majority of patients sought treatment either at a clinic (56%) or a pharmacy (15%). The frequency and duration of ALA was higher in patients with larger circumference of the leg measured below the level of the knees (χ^2^ = 5.94, p<0.05 and χ^2^ = 6.67, p<0.01, respectively). Neither the frequency of attacks nor the duration of illness were significantly associated with age, sex, or age of onset of the illness.

Table 3 shows parameters used for clinical characterization. Mossy lesions were observed in 175 (53%) patients. It was more common among males (64%) than females (49%), and the difference was statistically significant (OR = 1.89, 95% CI = 1.15–3.11, p = 0.011). Open wounds were present in 24 (7.2%) patients and were more common among patients that had mossy lesions than those without (12.6% vs. 1.3%, respectively; χ^2^ = 15.6, p<0.001).

**Table 3:** Clinical characterization of feet and legs of patients.

Feature	Category	Male	Female	Overall
Mossy lesion (n = 331)	Present	59 (64.0)	116 (49.0)	175 (53.0)
	Absent	33 (36.0)	123 (51.0)	156 (47.0)
Open wound (n = 333)	Present	7 (8.0)	17 (7.0)	24 (7.0)
	Absent	85 (92.0)	224 (93.0)	309 (93.0)
Right leg circumference (cm) (n = 303)	21–25	8 (9.0)	18 (8.0)	26 (9.0)
	26–36	76 (86.0)	185 (86.0)	262 (87.0)
	37–47	4 (5.0)	10 (5.0)	14 (5.0)
Left leg circumference (cm) (n = 296)	21–25	9 (11.0)	20 (9.0)	29 (10.0)
	26–36	72 (87.0)	178 (84.0)	250 (85.0)
	37–47	2 (2.0)	15 (7.0)	17 (6.0)

### Factors related to footwear and personal hygiene

Respondents were asked about their experience of and attitudes towards footwear and personal hygiene, which are central to prevent, treat and control disease progression.

The majority of patients reported that they had no problem finding enough water and that it took an average of 10 minutes' walk to reach a water source. Two-thirds of patients reported washing their feet at least once per day, and 58% said they washed their feet with soap daily.

Of the study participants in the detailed study, 303 (96%) had worn shoes at least once in their life. The mean age when patients started wearing shoes was 23 years (±15.9), a time often coinciding with the onset of signs and symptoms of podoconiosis. The experience of wearing shoes did not vary between males and females. However, the type and quality of shoe worn varied, more males than females wearing the better quality and more expensive leather shoes (19.6% vs. 8.7%, χ^2^ = 7.4, p = 0.007). The times when people walked barefoot were in the field (22%), during rainy season (13%) and at home (11%) (Table 4).

**Table 4:** Factors related to footwear and personal hygiene.

Feature	Category	Male	Female	Overall
Access to water for washing feet	Perceived to have access to enough water (n, %)	88 (98.0)	230 (95.0)	318 (95.5)
	Minutes' walk to access water for washing (mean, s.d.)	9.5 (7.0)	10.2(9.0)	10.0 (8.5)
Foot washing habit	Practice of washing feet at least once per day (n, %)	86 (94.0)	227 (95.0)	313 (94.8)
	Frequency of washing feet per day (mean, s.d.)	1.7 (0.8)	1.7 (0.8)	1.7 (0.8)
	Frequency of washing feet with soap per week (mean, s.d.)	4.7 (2.7)	5.2 (3.4)	5.1 (3.2)
	Practice of washing feet with soap daily (n, %)	43 (50.0)	139 (61.0)	182 (58.2)
Footwear history	Has ever worn footwear (n, %)	88 (98.0)	215 (95.0)	303 (96.0)
	Age footwear first worn (mean, s.d.)	22.8(15.3)	22.9 (16.3)	22.9 (15.9)
	Worn footwear at time of interview (n, %)	85(94.0)	218 (90.5)	303 (91.0)
	Pairs of shoes owned (mean, s.d.)	1.8 (0.92)	1.7(0.85)	1.7 (0.87)
	Pairs of shoes needed per year (mean, s.d.)	2.9 (0.9)	2.8 (0.9)	2.8(0.9)
Type of footwear at time of interview (n, %)	Hard plastic	40 (43.5)	78 (32.4)	118 (35.4)
	EVA	21 (22.8)	72 (29.9	93 (27.9)
	Canvas	6 (6.5)	44 (18.3)	50 (15.0)
	Leather	18 (19.6)	21 (8.7)	39 (12.0)
	Barefoot	7 (7.6)	23 (9.5)	30 (9.0)
Situations when shoes are not worn (n, %)	In the field	20 (21.7)	41 (16.7)	61 (18.2)
	During rainy season	14 (15.2)	31(12.7)	45 (13.4)
	At home	11 (12.0)	26 (10.6)	37 (11.0)
	Never walks barefoot	62 (67.4)	159 (64.9)	221 (66.0)

## Discussion

The present study showed that podoconiosis is a problem of public health importance in Gulliso *woreda*, west Ethiopia. The prevalence of podoconiosis in Gulliso *woreda* (2.8%) is lower than reports from Wolaita zone, southern Ethiopia (5.5%) [4]. One of the limitations of our study is that it reported only cases of podoconiosis with overtly swollen legs. In addition, cases were examined only when they were reported by the head of the household. Given the stigma associated with podoconiosis, a number of patients may not have shown themselves to the study team. This may have resulted in an underestimate of the prevalence of podoconiosis. Moreover, the study *woreda* has relatively better access to education and health care, and is not likely to represent the most remote *woredas* in the zone or region, where higher prevalence rates are expected. It is possible that disease incidence has declined with better education, more shoe wearing and access to health care when compared to the other study areas. Recent studies also indicated the role of genetic factors in podoconiosis [23], and differences in disease prevalence among various population groups may be due to differences in frequency of genetic variants that confer susceptibility to podoconiosis. The prevalence of the disease in the *woreda* is still higher than that of all forms of tuberculosis (estimated to be 579 per 100,000 population for Ethiopia) [24] and HIV/AIDS (2.0% in Oromia region) [25]. As reported in other studies, the disease starts in the second and third decades of life and its prevalence and severity increases up to the 6^th^ decade of life, corroborating the suggestion that podoconiosis is a chronic condition that disables but rarely kills [16]. The prevalence of podoconiosis was much lower in villages dominated by settler populations. An epidemiological study in resettlement schemes of west Ethiopia showed that prevalence of podoconiosis is higher in indigenous people than in settlers (9.0% vs. 5.0%). People from non-endemic areas develop disease on average 9 years after being moved to an endemic area [21]. Moreover, observations in our survey showed that the *kebeles* with settler populations had higher use of footwear, better personal hygiene practices and a relatively advanced standard of living.

Interestingly, the prevalence of podoconiosis was higher among females than males similar to the studies in Ocholo (1∶4.2) [26], Wolaita (1∶1.4) [14] and Pawe (1∶1.4) [27]. An epidemiological study in settler populations in Keffa region [21] showed male predominance. Our survey also showed that in the two *kebeles* with settler populations the prevalence was slightly higher among males than females, but the difference was not statistically significant. In contrast, a more recent community based survey in Wolaita zone reported that the prevalence in males and females is similar [4]. The study also reported that 10% of women were not willing to be examined due to modesty, and this may have underestimated the prevalence in women. Our survey showed that early stages of podoconiosis were more common in women and severe forms of the disease and mossy lesions were more prevalent in men. This may be because females look for help earlier, as appearance constitutes a greater motivation for them. Moreover, women may have a better access to rinsing water during their routine activities in the household such as fetching water and washing clothes. This curbs progression of the disease to the advanced stages. Men may be indifferent to the condition initially, so do not look for care and do not look after the leg. Once the disease becomes severe they will also admit to having the condition.

One of the most important findings of this study is the high frequency of acute episodes of adenolymphangitis in 97% of the study population. Most patients sought medical treatment for acute episodes. Many described the occurrence of monthly episodes as coming always during ‘*chagin*o’, the local term to describe the time when the moon is absent. The study documented frequent acute attacks among patients, probably more frequent than those of malaria which is currently declining in the *woreda*. The associated symptoms and the subsequent exhaustion that lasts for days [13] cause serious morbidity and long absence from productive work. This presents further evidence of the widely held opinion that podoconiosis aggravates poverty due to working days lost (almost one month/year) and expenditure on health care, often ineffective. The incidence of adenolymphangitis and the resulting incapacitation found in our study was higher than that reported for filariasis in southeast Tanzania [28], south India [29], and Papua New Guinea [30]. Acute attacks of ALA were significantly positively associated with leg circumference. The larger the swelling, the less likely the person to find adequate footwear, leaving the legs more exposed to soil and predisposed to acute attacks.

The socio-economic impact of the disease is high. Of 10 patients, nine belonged to the economically active age group. Previous studies also showed the huge economic burden of podoconiosis [17], and high prevalence of the disease among the economically active age group [4]. Over half of the study subjects in our survey were uneducated, the rate being even higher among females. Moreover, the proportion of unmarried women among all patients was significantly higher than the proportion for rural Oromia region (19.2% vs. 6.1%, χ^2^ = 12.5, p<0.001), indicating stigmatisation [31]. Previous studies demonstrated several manifestations of social stigma among podoconiosis endemic communities [18], [32]. The disease leads to social exclusion of individuals and their families, e.g. school dropout, lack of marriage prospects, exclusion from community events. Social exclusion also leads to the invisibility of podoconiosis. The belief that effective treatment does not exist may discourage patients and health workers. These may be some of the factors that help explain the lack of attention paid to this important and common disease by researchers, policy makers, and health professionals.

The World Health Organization defines access to *drinking* water as the availability of at least 20 litres of water per person per day within a round trip walking distance of 30 minutes [33]. Accordingly, the patients in our survey are at a reasonable distance from the source of water (20 minutes round trip), but may not have access to adequate water. The use of protective shoes, combined with consistent washing with soap and water was uncommon among study subjects. There were also gender disparities in the quality of shoes worn. Disproportionately fewer females wore the better quality leather shoes compared to males.

In conclusion, we have documented high podoconiosis prevalence, frequent adenolymphangitis and high disease-related morbidity in west Ethiopia. This was evidenced by the high prevalence of the disease, incidence of adenolymphangitis and the associated socio-economic impact. Interventions must be developed to raise awareness of podoconiosis, to prevent, treat and control the disease. The higher prevalence among women implies that women's development and empowerment programs that operate in west Ethiopia must embrace podoconiosis prevention and control programs. Furthermore, podoconiosis control and prevention programs must give additional attention to women to treat and rehabilitate them so that they can resume important social and economic roles in the community.

## Supporting Information

Checklist S1 STROBE checklist.
(DOC)
