# Supplementary material for: Burden of Podoconiosis in Poor Rural Communities in Gulliso woreda, West Ethiopia
Source: PLoS Negl Trop Dis. 2011 Jun 7;5(6):e1184. doi: 10.1371/journal.pntd.0001184 (PMC3110157; doi:10.1371/journal.pntd.0001184)
Supplement: Checklist S1 — STROBE checklist. (DOC) [file pntd.0001184.s001.doc]

STROBE Statement—Checklist of items that should be included in reports of ***cross-sectional studies***

|  | Item No | Recommendation |
| --- | --- | --- |
| **Title and abstract** | 1 | (*a*) Indicate the study’s design with a commonly used term in the title or the abstract  A cross-sectional study (abstract) |
|  |  | (*b*) Provide in the abstract an informative and balanced summary of what was done and what was found  Done |
| Introduction | | |
| Background/rationale | 2 | Explain the scientific background and rationale for the investigation being reported  Done, 6^th^ statement in the last paragraph of the introduction |
| Objectives | 3 | State specific objectives, including any prespecified hypotheses  Done, last statement in the last paragraph of the introduction |
| Methods | | |
| Study design | 4 | Present key elements of study design early in the paper  Done, pp7 |
| Setting | 5 | Describe the setting, locations, and relevant dates, including periods of recruitment, exposure, follow-up, and data collection  Done, under ‘study area’ on pp6 |
| Participants | 6 | (*a*) Give the eligibility criteria, and the sources and methods of selection of participants  Done, under ‘study design and sampling’ on pp7 |
| Variables | 7 | Clearly define all outcomes, exposures, predictors, potential confounders, and effect modifiers. Give diagnostic criteria, if applicable  Done, second paragraph under ‘data collection’ on pp8 and ‘study design and sampling’ on pp7 |
| Data sources/ measurement | 8* | For each variable of interest, give sources of data and details of methods of assessment (measurement). Describe comparability of assessment methods if there is more than one group  Done, under ‘data collection, pp9 |
| Bias | 9 | Describe any efforts to address potential sources of bias  Trained nurses recruited patients. A published and validated clinical staging system was used to stage disease. Simple random sampling was used to select kebeles. |
| Study size | 10 | Explain how the study size was arrived at  Done, pp7. All households in the rural 26 kebeles were surveyed. All patients identified in the survey were recorded. |
| Quantitative variables | 11 | Explain how quantitative variables were handled in the analyses. If applicable, describe which groupings were chosen and why  Described under ‘Data analysis’ |
| Statistical methods | 12 | (*a*) Describe all statistical methods, including those used to control for confounding |
|  |  | (*b*) Describe any methods used to examine subgroups and interactions |
|  |  | (*c*) Explain how missing data were addressed |
|  |  | (*d*) If applicable, describe analytical methods taking account of sampling strategy |
|  |  | (*e*) Describe any sensitivity analyses  Described under ‘Data analysis’ |
| Results | | |
| Participants | 13* | (a) Report numbers of individuals at each stage of study—eg numbers potentially eligible, examined for eligibility, confirmed eligible, included in the study, completing follow-up, and analysed |
|  |  | (b) Give reasons for non-participation at each stage  N/A |
|  |  | (c) Consider use of a flow diagram  Ethical and legal issues related with inclusion of children under 18 years of age is described under ‘Ethics statement’.  Numbers of individuals studied included under ‘study population and disease related characteristics’ |
| Descriptive data | 14* | (a) Give characteristics of study participants (eg demographic, clinical, social) and information on exposures and potential confounders  Done, under Table 1 |
|  |  | (b) Indicate number of participants with missing data for each variable of interest  Indicated in each Table as ‘n’ |
| Outcome data | 15* | Report numbers of outcome events or summary measures  Table 3, and under ‘features of disease’ |
| Main results | 16 | (*a*) Give unadjusted estimates and, if applicable, confounder-adjusted estimates and their precision (eg, 95% confidence interval). Make clear which confounders were adjusted for and why they were included |
|  |  | (*b*) Report category boundaries when continuous variables were categorized |
|  |  | (*c*) If relevant, consider translating estimates of relative risk into absolute risk for a meaningful time period  95% CI given in tables and in the text. Category boundaries given to continuous variables such as age, leg circumference in Tables 1-3 |
| Other analyses | 17 | Report other analyses done—eg analyses of subgroups and interactions, and sensitivity analyses  N/A |
| Discussion | | |
| Key results | 18 | Summarise key results with reference to study objectives  Done, paragraph 1 |
| Limitations | 19 | Discuss limitations of the study, taking into account sources of potential bias or imprecision. Discuss both direction and magnitude of any potential bias  Done, paragraph 1 |
| Interpretation | 20 | Give a cautious overall interpretation of results considering objectives, limitations, multiplicity of analyses, results from similar studies, and other relevant evidence  Done, Paragraphs 2-5 |
| Generalisability | 21 | Discuss the generalisability (external validity) of the study results  Last paragraph |
| Other information | | |
| Funding | 22 | Give the source of funding and the role of the funders for the present study and, if applicable, for the original study on which the present article is based  Done, under ‘financial disclosure’ |

*Give information separately for exposed and unexposed groups.

**Note:** An Explanation and Elaboration article discusses each checklist item and gives methodological background and published examples of transparent reporting. The STROBE checklist is best used in conjunction with this article (freely available on the Web sites of PLoS Medicine at http://www.plosmedicine.org/, Annals of Internal Medicine at http://www.annals.org/, and Epidemiology at http://www.epidem.com/). Information on the STROBE Initiative is available at www.strobe-statement.org.
